# Educational performance of TikTok short videos for age related macular degeneration and its link to user engagement

**DOI:** 10.3389/fmed.2025.1751545

**Published:** 2026-01-12

**Authors:** Ligang Jiang, Xin Jiang, Xia Shi, Xinya Hu, Chunyan Song, Weihua Yang, Yuhua Tong

**Affiliations:** 1 Department of Ophthalmology, Quzhou Affiliated Hospital of Wenzhou Medical University, Quzhou People’s Hospital, Quzhou, Zhejiang, China; 2 Quzhou College of Technology, Quzhou, Zhejiang, China; 3 Department of Ophthalmology, Quzhou Kecheng People’s Hospital, Zhejiang Cancer Hospital Quzhou Branch, Zhejiang Hospital Quzhou Branch, Quzhou, Zhejiang, China; 4 Shenzhen Eye Hospital, Shenzhen Eye Medical Center, Southern Medical University, Shenzhen, China

**Keywords:** age-related macular degeneration, health education, social media, TikTok, video quality

## Abstract

**Background:**

To systematically evaluate the information quality, reliability, and content characteristics of short videos related to age-related macular degeneration on the Chinese mainland version of TikTok, filling the research gap in this field and providing references for ophthalmic health education and platform information governance.

**Methods:**

This cross-sectional study was conducted on October 15, 2025, by searching the keyword “年龄相关性黄斑变性” on TikTok. The top 200 videos under “comprehensive ranking” were screened, and 196 videos meeting the eligibility criteria were ultimately included. Content integrity was evaluated following the American Academy of Ophthalmology guidelines and the Goobie framework. Video quality was assessed using the DISCERN instrument and the PEMAT-A/V tool. Statistical analyses were performed in IBM SPSS Statistics 27.0, with inter-rater reliability measured by the intraclass correlation coefficient. Differences among groups and associations between variables were examined using ANOVA and correlation analysis.

**Results:**

The overall quality of the included videos was moderate, with a median DISCERN tool score of 48.00 and a median Overall quality score of 3.00. The Understandability score was relatively high (median 84.62%), whereas the Actionability score was lower (median 75.00%). Videos uploaded by the Non-Profit group showed the highest quality (mean DISCERN tool score 59.13 ± 2.50), followed by the Medical group. The Non-Medical and For-Profit groups demonstrated the lowest quality, with statistically significant differences among groups (*P* < 0.05). Quality metrics were moderately positively correlated with user engagement metrics. The correlation coefficients between reliability and engagement were *r* = 0.48–0.54 (*P* < 0.05). Video duration showed a mild positive correlation with both quality and engagement (*r* = 0.23–0.30, *P* < 0.05). Inter-rater reliability was good (intraclass correlation coefficient = 0.839–0.947, *P* < 0.001).

**Conclusion:**

Age-related macular degeneration videos on TikTok showed moderate overall quality, with content emphasizing clinical concerns but neglecting basic knowledge. Information quality varied by uploader source, with non-profit organizations and medical professionals providing most high-quality content. Higher-quality videos tended to receive greater user engagement, suggesting that platform algorithms may preferentially spread better educational material. These findings provide empirical support for improving science communication on this disease and strengthening information quality management on digital platforms.

## 1 Introduction

Age-related macular degeneration (AMD) is a chronic, progressive disorder of the macula and one of the leading causes of irreversible vision loss in older adults worldwide (1, 2). Recent projections suggest that the number of people affected by AMD will reach approximately 288 million by 2040 (3), imposing a substantial burden on health-care systems and patients’ daily functioning (4). Because AMD primarily impairs central vision, patients may struggle with reading, driving, and facial recognition, which undermines independence and is associated with a higher risk of depressive symptoms (5, 6). Given the importance of early intervention in slowing disease progression, improving public awareness of AMD risk factors, symptoms, and evidence-based management is essential (7–9).

In parallel, social media has reshaped how the public acquires and shares health information. Short-video platforms such as TikTok, with algorithmically curated feeds and highly visual, narrative content, have rapidly become important channels for medical communication (10–13). However, because content is user-generated and rarely peer-reviewed, misinformation and incomplete messages are common and may adversely affect health literacy and care-seeking behavior (14–17). While previous studies have assessed the quality of ophthalmic videos, such as glaucoma and cataract content on traditional platforms like YouTube (18–20), there is still a lack of systematic evaluation of AMD-related information on TikTok, particularly within the Chinese mainland context. To address this gap, the present study used established ophthalmic guidelines and validated quality instruments to evaluate the information quality, reliability, and content characteristics of AMD-related TikTok videos and to explore their relationship with user engagement.

## 2 Materials and methods

### 2.1 Study framework and search protocol

This study adopted a cross-sectional design. A comprehensive video search was performed on October 15, 2025, on the TikTok platform (Douyin, the mainland Chinese version). To minimize recommendation bias caused by platform algorithms, browser history and cookies were cleared before the search, which was conducted without logging into any user account. The following Chinese keyword was used: “年龄相关性黄斑变性.” The personalized recommendation filter was disabled during the search to obtain the most objective results possible.

To ensure analytical feasibility while capturing the most relevant and frequently recommended content for this keyword, we predefined the sample and limited screening to the first 200 videos ranked under “comprehensive sorting.” This sample size balances coverage of the AMD-related information space on TikTok with the workload of duplicate rating and detailed scoring for each clip. After preliminary screening of these 200 videos, those meeting the eligibility criteria were included in the final analysis.

### 2.2 Inclusion and exclusion criteria

The inclusion criteria were as follows: (1) the video content was directly related to the etiology, symptoms, diagnosis, treatment, or prevention of AMD; (2) the video was presented in Chinese; and (3) the video was fully accessible for playback. The exclusion criteria were as follows: (1) videos irrelevant to the topic; (2) duplicate content; (3) videos of insufficient quality for evaluation; (4) advertisements created solely for product promotion; and (5) user comments or non–primary video content. Two researchers with medical backgrounds independently screened the videos, and any discrepancies were resolved through discussion with a third senior investigator who made the final decision.

### 2.3 Data extraction and uploader classification

For each included video, a standardized data extraction form was designed, and information was independently collected by two researchers. The extracted data included: (1) basic video characteristics, such as URL, upload date, duration, number of likes, comments, favorites, and shares; and (2) information on the video uploader. Based on the uploader’s profile, video content, and verification status, uploaders were categorized into four groups:

(i)    Medical group: certified individual health-care professionals (e.g., ophthalmologists, other physicians, or nurses) whose profiles clearly emphasized a personal professional identity;
(ii)    Non-Medical group: non-medical users, including patients, relatives, or members of the general public without verifiable medical credentials;
(iii)    For-Profit group: commercial entities such as private medical institutions, pharmaceutical or medical device companies, and commercial media accounts whose primary purpose appeared to be marketing or promotion;
(iv)    Non-Profit group: non-profit organizations, including public health-care institutions (e.g., tertiary public hospitals), governmental health authorities, and news or public-service organizations.

In practice, accounts were coded as Non-Profit when the profile explicitly represented an institution (e.g., hospital or health agency name in the account title, official logo, institutional verification badge, or descriptions such as “official account of XX Hospital”). Accounts were coded as Medical when they primarily highlighted a named clinician and used personal pronouns (e.g., “I,” “my clinic”), even if a hospital affiliation was listed in the profile. In the Chinese context, public hospitals and governmental agencies were treated as Non-Profit organizations, whereas private clinics and commercial health companies were categorized as For-Profit. Ambiguous accounts were jointly discussed by the two raters, and disagreements were resolved by a third senior ophthalmologist.

### 2.4 Video content assessment and quality tools

To ensure the scientific rigor of the evaluation, this study adopted the Age-Related Macular Degeneration Preferred Practice Pattern issued by the American Academy of Ophthalmology (AAO) as the content evaluation standard (21). The content completeness was assessed according to the educational video evaluation framework proposed by Goobie et al. (22), covering six core aspects of AMD: (1) definition, (2) classification, (3) symptoms, (4) risk factors, (5) diagnostic methods, and (6) treatment and management.

Video quality was evaluated using two validated tools: The DISCERN instrument, which consists of 16 questions, is designed to assess the quality of consumer health information regarding treatment options and is considered the gold standard for evaluating the reliability of health information. Each question is rated on a 5-point scale, with a total score ranging from 16 to 80, where higher scores indicate better quality (23, 24). The Patient Education Materials Assessment Tool for Audiovisual Materials (PEMAT-A/V) consists of 17 items divided into two domains: Understandability and Actionability, rated as “agree,” “disagree,” or “not applicable” (25, 26). Prior to the formal evaluation, all raters received standardized training. A random sample of 20 videos was selected for a pilot evaluation to ensure inter-rater reliability, and the intraclass correlation coefficient (ICC) among raters was subsequently calculated.

### 2.5 Statistical analysis

All statistical analyses were performed using IBM SPSS Statistics version 27.0 (IBM Corp., Armonk, NY, United States). The Shapiro–Wilk test was employed to examine the normality of data distributions. Continuous variables were summarized as mean ± standard deviation (mean ± SD) for approximately normally distributed variables and as median (P25, P75) for non-normally distributed variables. Inter-rater consistency between the two evaluators was assessed using the intraclass correlation coefficient (ICC). Differences in quality scores among the four uploader groups were first examined using one-way analysis of variance (ANOVA). When the homogeneity of variance assumption was violated, Welch’s ANOVA or the Kruskal–Wallis test was used instead. Where global tests were statistically significant, pairwise *post hoc* comparisons were conducted using Bonferroni-adjusted *t*-tests (for ANOVA) or Dunn–Bonferroni tests (for Kruskal–Wallis) to control for type I error. Correlations between video characteristics and quality scores were examined using Pearson’s correlation coefficient for approximately normally distributed variables and Spearman’s rank correlation coefficient for non-normally distributed variables. In particular, engagement metrics, which showed markedly skewed distributions, were analyzed using Spearman’s rank correlations. All statistical tests were two-sided, and a *P* < 0.05 was regarded as statistically significant.

## 3 Results

### 3.1 Video identification and screening

A total of 200 videos were initially retrieved through the preliminary search. After removing three duplicate videos, 197 videos remained for preliminary screening. Screening based on titles and video content led to the exclusion of one video that was either unrelated to the topic of AMD or purely promotional. Ultimately, a total of 196 videos were included in the final analysis.

### 3.2 Uploader distribution and engagement metrics

**FIGURE 1:**
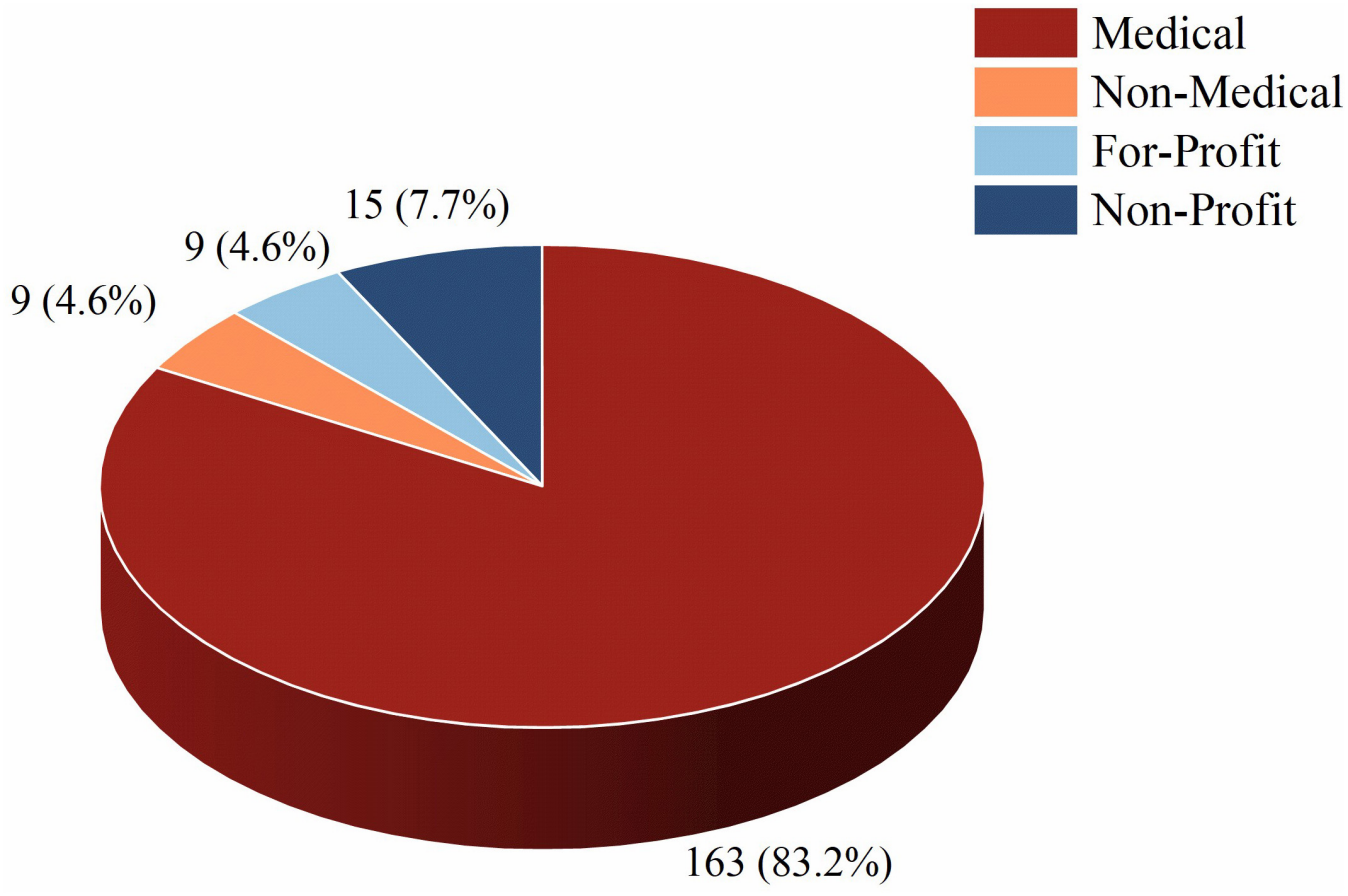
Proportion of videos from different uploader sources. The pie chart shows the percentage of videos uploaded by Medical, Non-Medical, For-Profit, and Non-Profit users among all included videos (*n* = 196).

Among the 196 included videos, the distribution of video uploaders was as follows: The Medical group uploaded the largest number of videos (*n* = 163, 83.2%), followed by the Non-Profit group (*n* = 15, 7.7%), and both the Non-Medical and For-Profit groups contributed nine videos each (4.6%). Details are shown in Figure 1.

Regarding engagement metrics, the mean number of likes was 2762.04 ± 10845.78, with a median of 325.00 (101.00, 996.25). The mean number of comments was 157.25 ± 561.55, with a median of 20.00 (8.00, 66.25). The mean number of favorites was 1113.52 ± 5106.81, with a median of 104.00 (34.25, 379.25). The mean number of shares was 1296.00 ± 7428.63, with a median of 68.50 (16.00, 287.00), and the mean video duration was 118.14 ± 146.46 s, with a median of 71.00 (47.00, 131.50). Details are provided in Table 1.

**TABLE 1:** Video information.

Variables	Average (SD)	M(P25, P75)
Likes	2762.04 ± 10845.78	325.00(101.00, 996.25)
Comments	157.25 ± 561.55	20.00(8.00, 66.25)
Favorites	1113.52 ± 5106.81	104.00(34.25, 379.25)
Shares	1296.00 ± 7428.63	68.50(16.00, 287.00)
Video duration	118.14 ± 146.46	71.00(47.00, 131.50)
Reliability	24.88 ± 4.39	25.00(23.00, 27.00)
Treatment choice	19.58 ± 3.02	20.00(19.00, 21.00)
Overall quality score	2.96 ± 0.93	3.00(2.00, 4.00)
DISCERN tool scores	47.42 ± 7.27	48.00(47.00, 50.00)
PEMAT-A/V understandability	81.48 ± 10.59	84.62(76.92, 84.62)
PEMAT-A/V actionability	73.30 ± 14.95	75.00(66.67, 75.00)

### 3.3 Thematic analysis by uploader source

**FIGURE 2:**
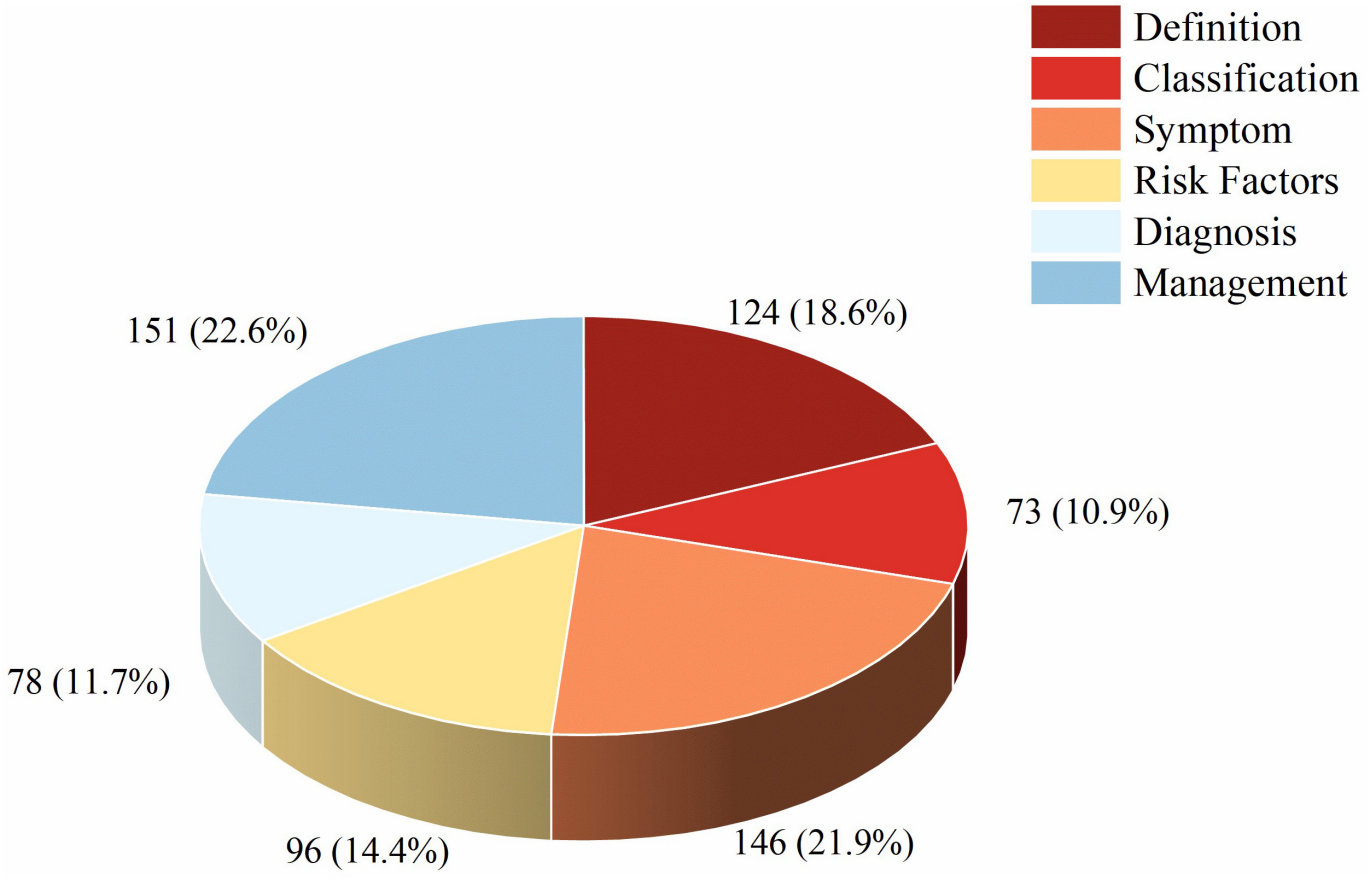
Distribution of video contents across AMD-related domains. The bar chart displays the number and percentage of videos covering each of the six content domains: definition, classification, symptoms, risk factors, diagnosis, treatment and management.

We analyzed the thematic content of 196 included videos, noting that many of them covered multiple topics simultaneously. As shown in Figure 2 and Table 2, the most frequently mentioned topic was Management (*n* = 151, 22.6%), followed by Symptoms (*n* = 146, 21.9%). In contrast, Classification (*n* = 73, 10.9%) and Diagnosis (*n* = 78, 11.7%) were the least frequently addressed. Definition and Risk Factors accounted for 18.6% (*n* = 124) and 14.4% (*n* = 96), respectively. These findings indicate that the videos tend to focus more on symptoms and management, which are of primary concern to patients, while professional definitions and diagnostic aspects are less frequently discussed.

**TABLE 2:** Content coverage of AMD-related TikTok videos across six core domains stratified by uploader type.

Core domain	Medical (*n* = 163)	Non-Medical (*n* = 9)	Non-Profit (*n* = 15)	For-Profit (*n* = 9)	Total (*n* = 196)
Definition	65.03% (106)	33.33% (3)	86.67% (13)	22.22% (2)	63.27% (124)
Classification	35.58% (58)	11.11% (1)	80.00% (12)	22.22% (2)	37.24% (73)
Symptom	78.53% (128)	22.22% (2)	86.67% (13)	33.33% (3)	74.49% (146)
Risk factors	50.92% (83)	11.11% (1)	73.33% (11)	11.11% (1)	48.98% (96)
Diagnosis	39.88% (65)	22.22% (2)	66.67% (10)	11.11% (1)	39.80% (78)
Management	77.30% (126)	44.44% (4)	93.33% (14)	77.78% (7)	77.04% (151)

**FIGURE 3:**
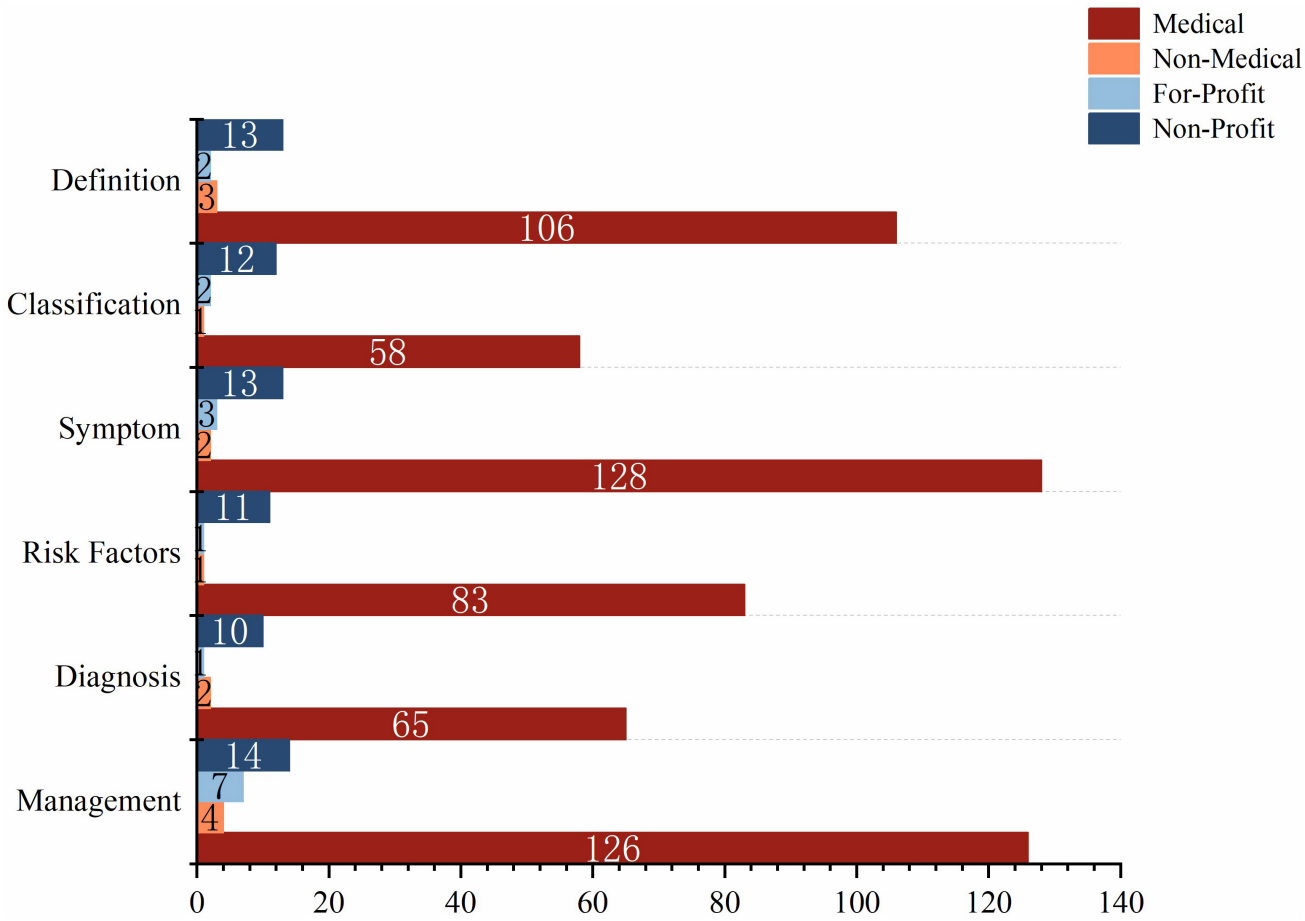
Proportion of content domains by uploader source. Stacked bars illustrate, for each uploader group (Medical, Non-Medical, For-Profit, and Non-Profit), the proportion of videos addressing each of the six AMD-related domains.

Regarding content sources, the Medical group was the predominant contributor across all thematic categories. For example, within the most popular Management topic, 126 videos (83.44%) originated from the Medical group, far exceeding other sources. Similarly, the Medical group also played a dominant role in the Symptom and Definition categories. The Non-Medical group showed a relatively sparse distribution of video content across topics. Videos from the For-Profit group primarily focused on Management, accounting for 77.78% of their uploads, while the Non-Profit group covered all six thematic aspects, with a minimum coverage rate of 66.67%, as shown in Figure 3.

### 3.4 Quality scores and between-group comparisons

**FIGURE 4:**
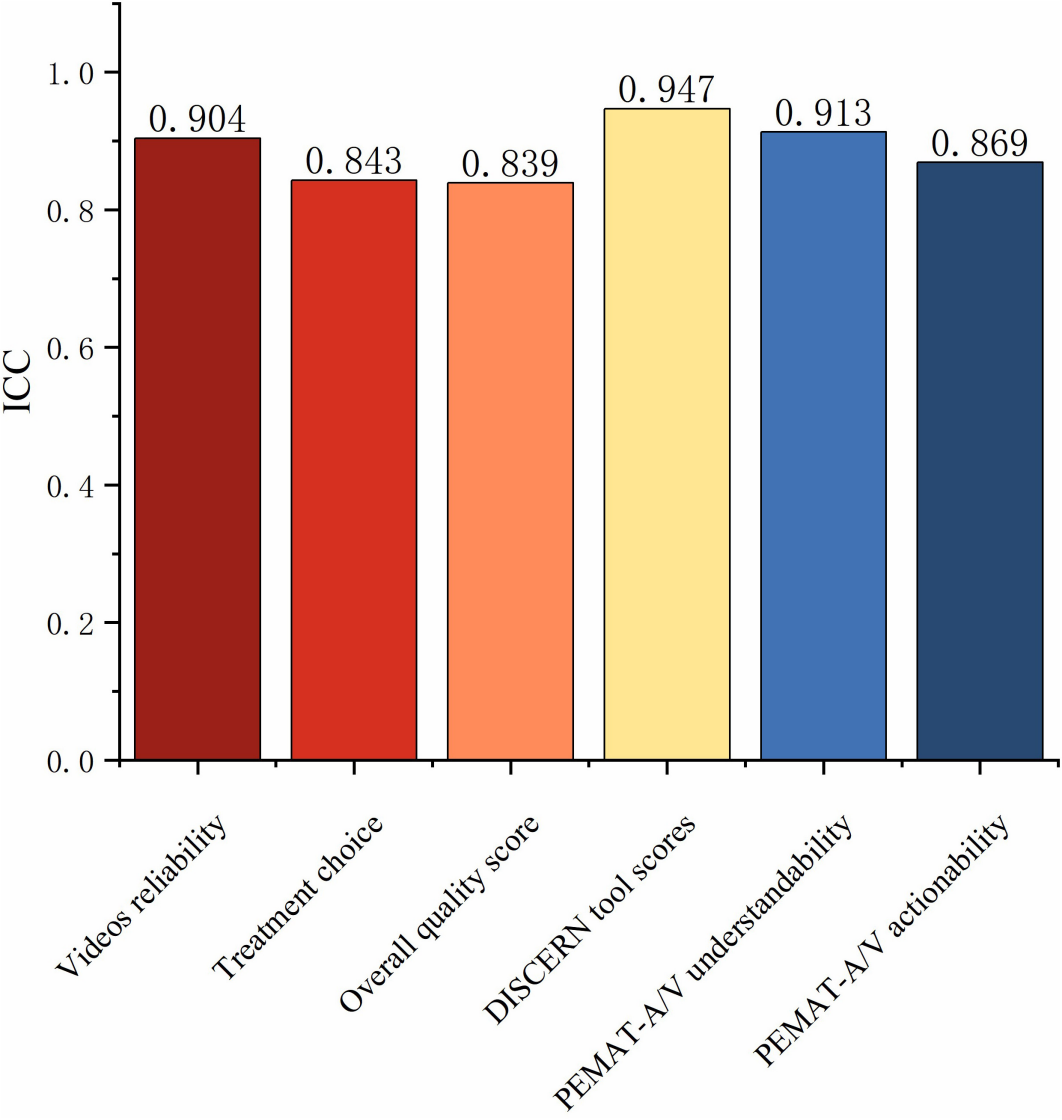
Inter-rater agreement for video quality assessments. ICCs between two independent raters are shown for Reliability, Treatment choice, DISCERN total score, Overall quality score, PEMAT-A/V Understandability, and PEMAT-A/V Actionability.

**FIGURE 5:**
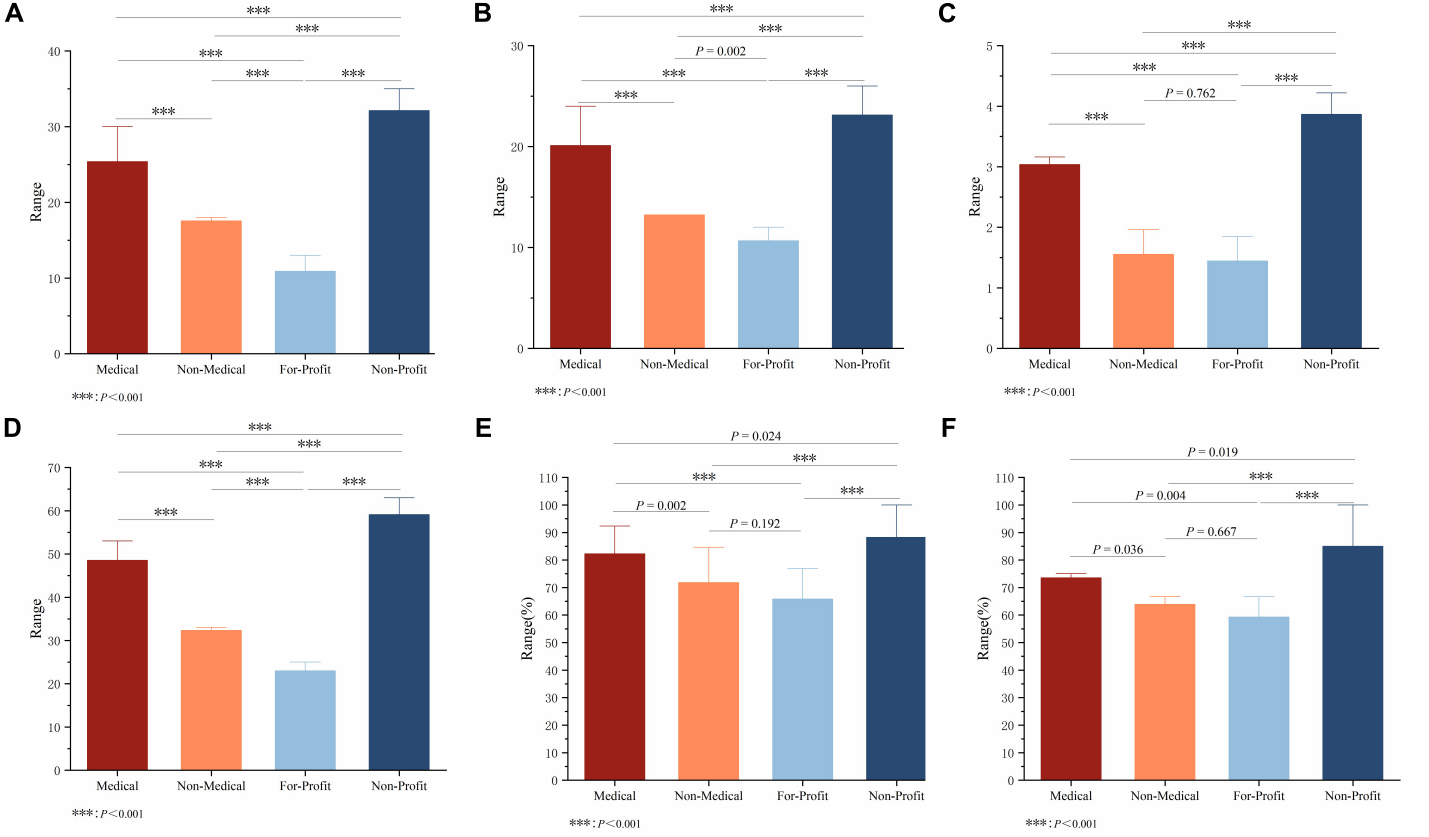
Comparison of video quality indicators across uploader sources. **(A)** Reliability, **(B)** Treatment choice, **(C)** Overall quality score, **(D)** DISCERN total score, **(E)** PEMAT-A/V Understandability, and **(F)** PEMAT-A/V Actionability were compared among the Medical, Non-Medical, For-Profit, and Non-Profit groups, ***P < 0.001.

For Reliability, Treatment choice, DISCERN tool scores, Overall quality score, Understandability, and Actionability, the ICCs between two raters were 0.904, 0.843, 0.839, 0.947, 0.913, and 0.869, respectively (*P* < 0.001) (Figure 4). The mean Reliability score for all included videos was 24.88 ± 4.39, with a median of 25.00, while the mean Treatment choice score was 19.58 ± 3.02, with a median of 20.00. The median Overall quality score of the videos was 3.00, with a mean of 2.96 ± 0.93, indicating a moderate overall quality. The DISCERN tool score had a median of 48.00 and a mean of 47.42 ± 7.27, also within the moderate range. However, the videos performed well in terms of Understandability (median = 84.62%), but scored slightly lower in Actionability (median = 75.00%) (Table 1). Significant differences among groups were observed in Reliability, Treatment choice, and DISCERN tool scores (*P* < 0.05) (Figures 5A,B,D). For Overall quality score, no significant difference was found between the Non-Medical and For-Profit groups (*P* = 0.762), whereas other intergroup comparisons revealed significant differences (P < 0.001) (Figure 5C).

In the intergroup comparison of Understandability, significant statistical differences were observed between the Medical and Non-Medical groups (*P* = 0.002), the Medical and Non-Profit groups (*P* < 0.001), the Non-Medical and Non-Profit groups (*P* < 0.001), and the For-Profit and Non-Profit groups (*P* < 0.001), whereas no significant difference was found between the Non-Medical and For-Profit groups (*P* = 0.192) (Figure 5E). In terms of numerical trends, the Non-Profit group exhibited the highest Understandability scores, followed by the Medical group, while the For-Profit group showed the lowest scores.

Regarding Actionability, the Non-Profit group again achieved the highest scores, followed by the Medical group, with the For-Profit group scoring the lowest. In the intergroup comparison of Actionability, significant differences were also found between the Medical and Non-Medical groups (*P* = 0.036), the Medical and For-Profit groups (*P* = 0.004), the Medical and Non-Profit groups (*P* = 0.019), the Non-Medical and Non-Profit groups (*P* < 0.001), and the For-Profit and Non-Profit groups (*P* < 0.001), whereas no significant difference was detected between the Non-Medical and For-Profit groups (*P* = 0.667) (Figure 5F).

Significant differences in video quality were observed across different uploader types. We compared video quality among four categories of uploaders. Results showed that videos uploaded by Non-Profit organizations achieved the highest scores across all quality dimensions, with a mean Reliability score of 32.13 ± 1.85, mean Treatment choice score of 23.13, median Overall quality score of 4.00, mean DISCERN tool scores of 59.13 ± 2.50, median PEMAT-A/V Understandability of 84.62%, and median PEMAT-A/V Actionability of 75.00%, all significantly higher than those of other groups (*P* < 0.001). The Medical group, as the dominant uploader category, ranked second in overall video quality, with generally above-average scores: median Reliability 25.00, median Treatment choice 20.00, median Overall quality score 3.00, median DISCERN tool scores 48, median Understandability 84.62%, and median Actionability 75.00%. In contrast, videos from the Non-Medical and For-Profit groups demonstrated the lowest quality. Notably, the For-Profit group had a median Overall quality score of only 1.00 and a median DISCERN tool score of just 22.00 (Table 3).

**TABLE 3:** Scores by different video sources.

Variables	Medical (*n* = 163)	Non-Medical (*n* = 9)	For-Profit (*n* = 9)	Non-Profit (*n* = 15)	*P*-value
Reliability (*n* = 196)	25.00 (24.00, 27.00)	17.56 ± 1.67	10.89 ± 1.62	32.13 ± 1.85	<0.001
Treatment choice (*n* = 196)	20.00 (19.00, 21.00)	13.00 (13.00, 13.50)	10.00 (10.00, 12.00)	23.13 ± 1.96	<0.001
Overall quality score (*n* = 196)	3.00 (2.00, 4.00)	2.00 (1.00, 2.00)	1.00 (1.00, 2.00)	4.00 (3.00, 4.00)	<0.001
DISCERN tool scores (*n* = 196)	48.00 (47.00, 50.00)	32.33 ± 2.00	22.00 (21.50, 25.0)	59.13 ± 2.50	<0.001
PEMAT-A/V understandability (%)	84.62 (76.92, 84.62)	71.80 ± 12.76	69.23 (57.70, 69.23)	84.62 (84.62, 92.31)	<0.001
PEMAT-A/V actionability (%)	75.00 (66.67, 75.00)	66.67 (50.00, 66.67)	66.67 (50.00, 66.67)	75.00 (75.00, 100.00)	<0.001

**FIGURE 6:**
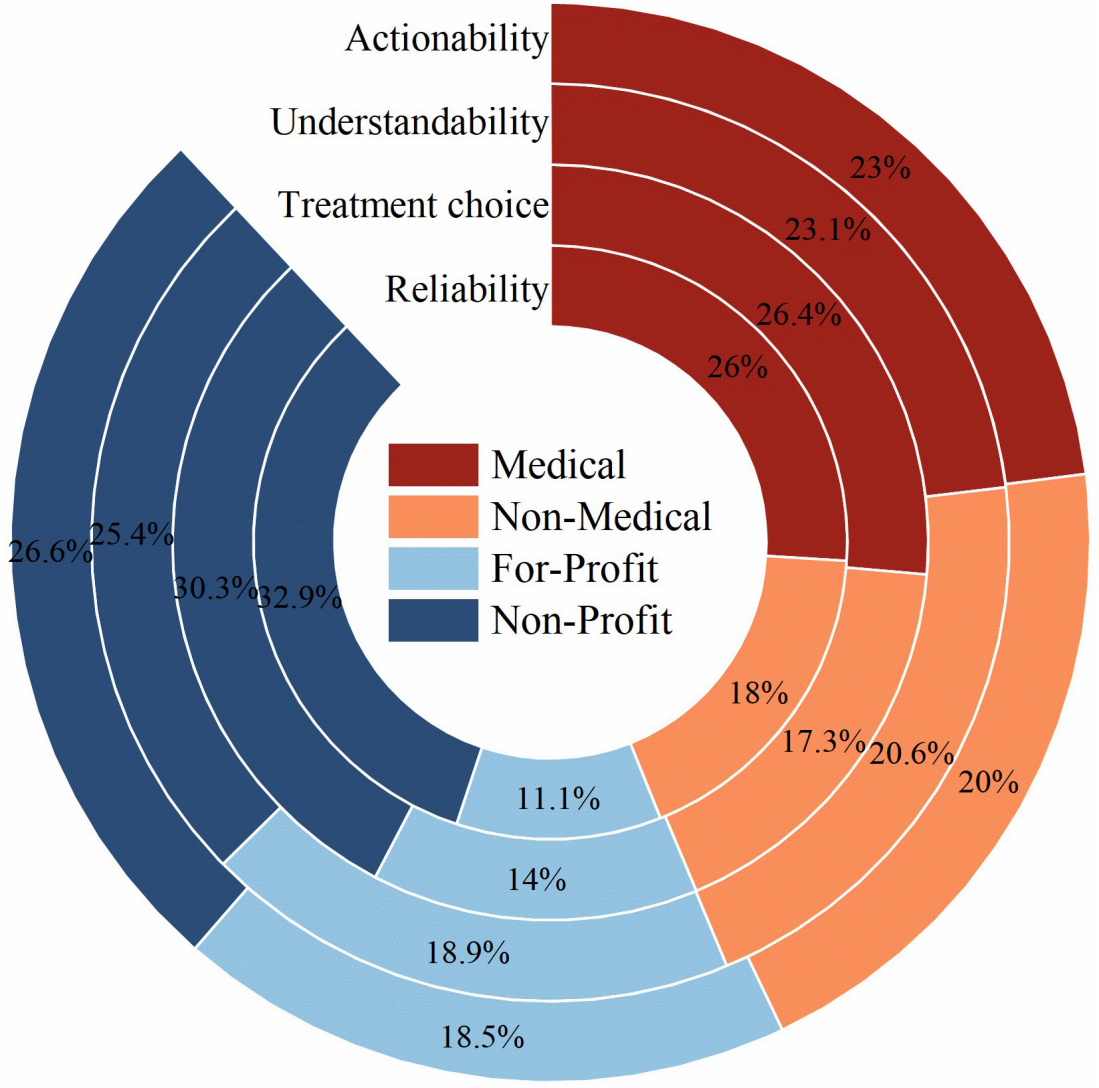
Contribution of each uploader group to overall quality metrics. Stacked bar charts show the percentage contribution of videos from each uploader source to total Reliability, Treatment choice, Understandability, and Actionability scores.

Regarding the proportion of Reliability scores, the Non-Profit and Medical groups accounted for 32.9 and 26.0%, respectively, whereas the Non-Medical and For-Profit groups contributed less, at only 18 and 11.1%. For Treatment choice, Non-Profit and Medical groups accounted for 30.3 and 26.4%, whereas Non-Medical and For-Profit groups accounted for only 17.3 and 14.0%. For Understandability, the Non-Profit and Medical groups contributed 25.4 and 23.1%, whereas the Non-Medical and For-Profit groups contributed only 20.6 and 18.9%. For Actionability, the Non-Profit and Medical groups accounted for 26.6 and 23.0%, while the Non-Medical and For-Profit groups contributed only 20.0 and 18.5%. Across these four dimensions, the Non-Profit and Medical groups consistently achieved higher performance levels (Figure 6).

### 3.5 Correlation between video quality assessment metrics

**FIGURE 7:**
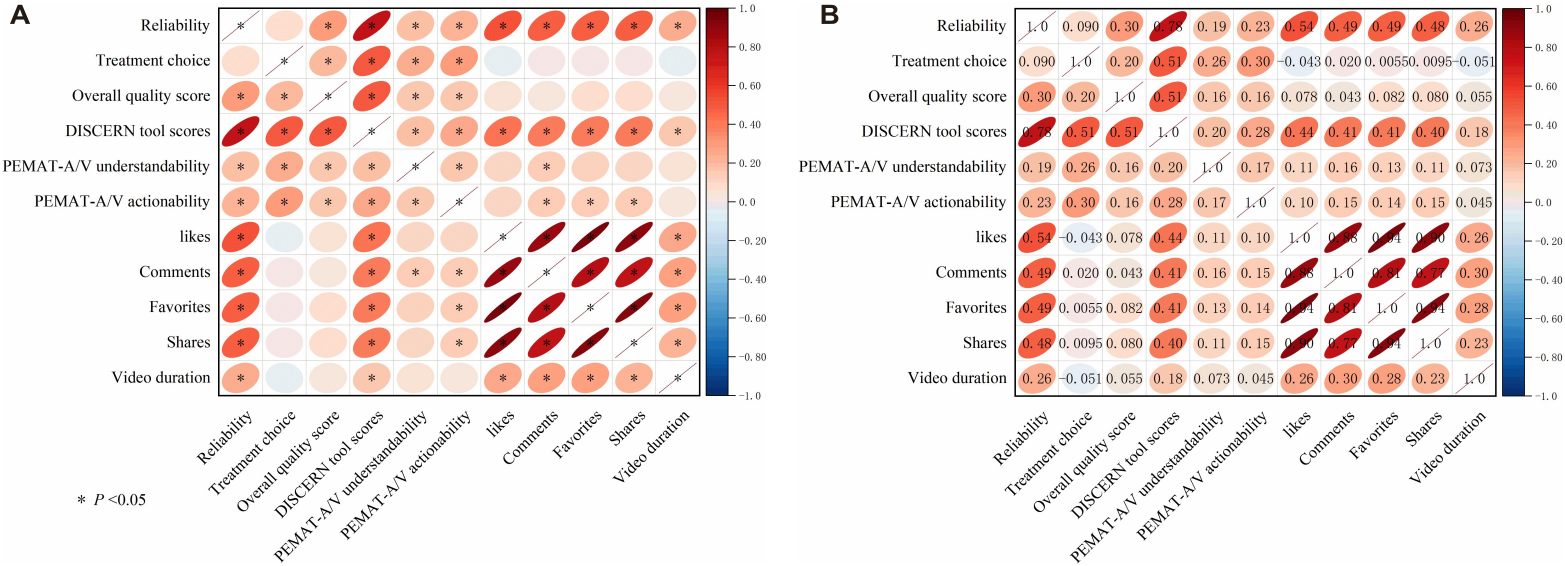
**(A)** Correlation analysis between video quality indicators and engagement metrics (correlation matrix heatmap). The heatmap depicts pairwise correlations among video quality indicators (Reliability, Treatment choice, Overall quality score, DISCERN tool scores, PEMAT-A/V Understandability, and PEMAT-A/V Actionability), engagement metrics (likes, comments, favorites, and shares), and video duration. **(B)** Matrix of correlation coefficients between video quality indicators and engagement metrics (numerical annotation). Each cell reports the corresponding correlation coefficient . Color and ellipse orientation/shape visualize the direction and magnitude of associations.

Significant positive correlations were generally identified among all video quality evaluation metrics (*P* < 0.05). DISCERN tool scores exhibited a strong positive correlation with Reliability (*r* = 0.78, *P* < 0.05) and moderate correlations with both the Overall quality score (*r* = 0.51, *P* < 0.05) and Treatment choice (*r* = 0.51, *P* < 0.05). This suggests that the DISCERN tool maintains strong alignment with the Reliability, Overall quality score, and Treatment choice domains when assessing the quality of information. Reliability demonstrated a moderate positive correlation with the Overall quality score (*r* = 0.30, *P* < 0.05), implying that higher reliability is generally accompanied by a higher overall quality rating; however, this relationship was weaker than the correlation between the DISCERN tool and Reliability. PEMAT-A/V revealed a weak correlation between Understandability and Actionability (*r* = 0.17, *P* < 0.05). Both metrics showed only weak-to-moderate positive correlations with DISCERN tool scores (*r* = 0.20 and *r* = 0.28, *P* < 0.05), suggesting that although PEMAT-derived Understandability and Actionability are related to DISCERN tool scores to some extent, their associations are weaker than those observed between DISCERN and other quality dimensions (Figure 7).

### 3.6 Correlation between video quality and engagement metrics

Reliability was moderately and positively associated with user engagement metrics, including likes, comments, favorites, and shares (*r* = 0.48–0.54, *P* < 0.05). Similarly, DISCERN tool scores were moderately correlated with these engagement metrics (*r* = 0.40–0.44, *P* < 0.05). However, correlations between PEMAT-A/V Understandability and Actionability and engagement metrics were relatively weak. Video duration showed a mild positive correlation with likes, comments, favorites, and shares (*r* = 0.23–0.30, *P* < 0.05). Moreover, video duration demonstrated weak positive correlations with Reliability (*r* = 0.26) and DISCERN tool scores (*r* = 0.18) (*P* < 0.05), but was not significantly correlated with Treatment choice (*r* = −0.051), Overall quality score (*r* = −0.055), Understandability (*r* = −0.073), or Actionability (*r* = −0.045) (*P* > 0.05) (Figure 7).

## 4 Discussion

This study systematically evaluated the information quality, reliability, and content characteristics of AMD-related short videos on the mainland China version of TikTok. Overall, the quality of these videos was moderate. Most clips focused on symptoms and treatment management, while disease classification and diagnostic information were less frequently addressed. Video quality differed significantly by uploader source. Videos from Non-Profit organizations achieved the highest quality scores, whereas the Medical group, despite being the largest contributor, produced content of only moderate quality. Additionally, video quality and reliability measures were positively correlated with user engagement, indicating that higher-quality content tended to attract more interaction.

The present study found that the overall quality of AMD-related videos on TikTok was at a moderate level. This finding shows both consistency and variation compared with previous studies investigating other social media platforms or different diseases. The consistency lies in the fact that “moderate quality” is a common feature of health information disseminated on social media. For instance, studies have reported (27) that YouTube videos concerning Behçet’s uveitis generally lack educational quality and reliability. Similarly, Bae et al. (28) reported heterogeneous video quality in their study of cataract surgery videos. Young et al. (29) found that publicly available videos on pediatric strabismus surgery contained generally low-quality information—only 59% of videos explained what strabismus is, and merely 25% accurately described the surgical procedure. Fazio et al. (30) analyzed TikTok videos on laser refractive surgery and found that, although videos created by medical professionals achieved slightly higher scores, the majority were of low reliability and content quality, containing a substantial amount of misleading information. Likewise, Cao et al. (19) reported similar findings in their assessment of cataract-related videos on TikTok, indicating that the overall video quality requires improvement. Consistent with previous research (31–33), videos published by Medical professionals were of significantly higher quality than those posted by Non-Medical users or For-Profit organizations. Moreover, content uploaded by Non-Profit organizations tended to be more reliable and of higher quality (34–36). These patterns suggest that moderate overall quality, higher scores for clinician- and institution-generated content, and lower scores for commercial or lay uploads are common features across ophthalmic conditions.

Beyond TikTok, YouTube has been more extensively studied as a source of ophthalmic video information. Evaluations of YouTube videos on AMD, optic neuritis, upper eyelid blepharoplasty and dry eye consistently report low-to-moderate DISCERN, JAMA and Global Quality Score values overall, with higher scores when videos are produced by ophthalmologists or professional institutions and poorer quality in patient- or commercially generated content (31, 37–39). Recent cross-platform analyses outside ophthalmology further suggest that, despite substantial variability, a markedly greater proportion of YouTube videos reach “useful” or “good quality” thresholds compared with TikTok, whereas TikTok clips tend to be shorter, more heterogeneous and more likely to contain misinformation (40). Taken together with our findings on AMD-related TikTok videos, these data indicate that both platforms face challenges in reliability and completeness. However, YouTube may currently provide more in-depth, guideline-concordant educational content, whereas TikTok’s short-form architecture and algorithm-driven amplification favor concise but less comprehensive messages. Future work should therefore compare AMD content across both platforms in parallel and explore how ophthalmologists can leverage the strengths of each ecosystem while actively mitigating their respective weaknesses when designing digital patient education.

Nevertheless, this study presents two particularly distinctive findings. First, medical professionals were identified as the main contributors of AMD-related videos on TikTok, differing from many prior studies in which non-professional users or commercial advertisements accounted for the majority of content (41–44). Despite their dominance, the videos produced by the Medical group were of significantly lower quality than those from Non-Profit organizations. This suggests that educational videos created by individual professionals may be less accurate or comprehensive compared with content produced by institutions employing formal review and quality control processes. Second, the most enlightening observation in this study is the strong positive correlation between video quality and user engagement. This implies that, on one hand, high-quality videos are more likely to be endorsed and shared by users (45); on the other hand, the algorithm-driven nature of TikTok may enhance the visibility of higher-quality medical content, thereby amplifying the educational impact of health information dissemination. This finding diverges from the results of several existing studies. Parmar et al. (20) reported no significant correlation between descriptive parameters and video quality. Kesimal et al. (38) similarly found no meaningful association between engagement metrics and the reliability or quality of optic neuritisiverges h conos on YouTube. In contrast, Haddad et al. (31) found that the quality of LASIK-related TikTok videos was positively correlated with engagement metrics, suggesting that audiences tend to interact more frequently with higher-quality content. Likewise, Barlas et al. (46) observed that YouTube videos on insulin resistance with higher quality were associated with greater views and user engagement. The favorable results of the present study may be explained by several potential factors: (1) AMD, as a specialized ophthalmic condition, attracts an audience that is relatively well educated and better equipped to evaluate information quality; and (2) TikTokovaluate information quality; and (2) Tike that is relatively tial fac were associated with great-quality content. These insights underscore the value of producing high-quality educational materials and may encourage medical professionals to actively contribute scientifically sound health information through social media.

The Non-Profit group achieved the highest scores across all quality assessment metrics, suggesting that its content may have undergone more rigorous peer review or internal auditing and placed greater emphasis on standardization in health education. Although Medical professionals were the primary contributors of popular science content, their overall video quality was only moderate, with a thematic focus on symptoms and management while inadequately addressing fundamental aspects such as classification and diagnosis. This may reflect the tendency of individual physicians to prioritize addressing patientsdiagnosis. This may reflect the tendency of individual physicians hematic focus on symp may have undergone more rigorous to disease knowledge (47). The videos scored relatively high in Understandability but low in Actionability, indicating that viewers or patients might understand the presented information yet remain uncertain about may hato do next.ing that viewers or patients might understand the presented informationto offer specific actionable guidance such as sfferto self-monitor using an Amsler gridable guidanto seek immediate medical attention.d Furthermore, this study found that slightly longer videos tended to provide more comprehensive and in-depth information, which was associated with higher reliability ratings and greater user engagement (48–50). This finding suggests that in producing health education content, it is inadvisable to excessively compress information; instead, an optimal balance should be achieved between conciseness and completeness (51).

From a practical standpoint, our findings offer several stakeholder-specific implications. For clinicians and health educators, the relatively low Actionability scores highlight the need to incorporate clear behavioral guidance into AMD-related videos, such as demonstrating how to use an Amsler grid, specifying red-flag symptoms that require urgent ophthalmic review, and directing viewers to reliable clinical services; doing so is likely to improve PEMAT-A/V Actionability scores. For TikTok and similar platforms, there is an opportunity to adjust recommendation and labeling mechanisms so that videos from verified healthcare professionals and Non-Profit organizations are more visible, while low-quality or overtly promotional content from For-Profit or non-medical accounts is flagged or down-ranked. Public health agencies and professional societies could also develop standardized AMD-related videos templates or checklists covering core domains (definition, classification, symptoms, risk factors, diagnosis and management) and make these resources available to uploaders to promote more complete, guideline-concordant content.

Although this study provides valuable insights, several limitations should be acknowledged. First, the sample of AMD-related TikTok videos was markedly unbalanced across uploader groups: Medical accounts dominated, whereas Non-Medical and For-Profit accounts contributed relatively few videos. As a result, estimates of quality for the smaller groups are less precise, and between-group comparisons should be interpreted with caution. Future studies should expand data collection, possibly across multiple time points and regions or in collaboration with other centers, to obtain larger and more balanced samples from under-represented uploader categories. Second, there is an inherent limitation related to the cross-sectional design. The data were collected through a one-time search conducted on October 15, 2025. Given TikTok’s rapidly evolving and highly dynamic nature, the list of the top 200 videos generated by the “comprehensive ranking” algorithm can change substantially within a short period; therefore, our findings represent only a snapshot of content at one time point and may not reflect longer-term trends. Third, the study is constrained by its narrow search strategy. Only the professional medical term “age-related macular degeneration” was used as the keyword, whereas members of the public may use non-technical or symptom-based keywords such as “macular disease,” “wavy vision,” or “blurred central vision in older adults.” Different search terms might yield videos with different sources, quality and thematic distributions. Fourth, subjectivity in assessment is an unavoidable limitation. Although we employed validated instruments (DISCERN and PEMAT-A/V), implemented standardized rater training and demonstrated good inter-rater reliability, scoring inevitably involves some degree of judgment. In future research we plan to broaden and compare search strategies, recruit multicenter research teams to increase sample diversity, and overcome the limitations of the cross-sectional design by adopting a longitudinal approach. By periodically tracking the quality, engagement and ranking dynamics of AMD-related videos over a 6–12 month period, we aim to better understand how content evolves over time on TikTok.

## 5 Conclusion

The present study revealed that videos concerning AMD on TikTok were generally of moderate quality. Non-Profit organizations produced the highest-quality content, while videos from Medical professionals ranked second. User engagement metrics were significantly and positively correlated with both reliability and overall quality, indicating that high-quality medical content tends to gain greater user recognition and reach. Moving forward, healthcare institutions and professionals should be encouraged to produce scientifically sound, easily understandable, and practically actionable health education videos to improve public awareness and facilitate the spread of credible medical information.

## Data Availability

The original contributions presented in the study are included in the article/supplementary material, further inquiries can be directed to the corresponding authors.
